# Risk assessment for yachting tourism in China using dynamic Bayesian networks

**DOI:** 10.1371/journal.pone.0289607

**Published:** 2023-08-23

**Authors:** Yunhao Yao, Xiaoxing Zhou, Merle Parmak

**Affiliations:** School of Public Administration & Humanities, Dalian Maritime University, Dalian, Liaoning, P.R. China; East China Normal University, CHINA

## Abstract

Scientific evaluation of yachting tourism safety risks (YTSRs) is crucial to reducing accidents in this sector. This paper is based on the data of 115 yachting tourism accidents in China’s coastal areas from 2008 to 2021. A fishbone diagram and the analytic hierarchy process (AHP) were used to identify the risk factors of yachting tourism from four aspects human, yachting, environmental, and management risk and to construct an evaluation index system. To perform dynamic evaluation, a dynamic evaluation model of YTSRs was built using dynamic Bayesian networks (DBN). The results indicate that human factors, such as the unsafe behavior of yachtsmen and tourists, are the primary risk factors; the risk is higher in summer than in winter, and the Pearl River Delta region has a greater risk of yachting tourism. It is suggested to improve the normal safety risk prevention and control system of yachting tourism, to advocate for multi-subject coordination and co-governance, and to improve the insurance service system so as to provide a guarantee for the safe and healthy development of yachting tourism in China. The findings provide theoretical and practical guidance for marine and coastal tourism safety management, as well as the prevention and control of YTSRs.

## Introduction

There is no uniform or precise definition of yachting tourism in the international context; for example, neither the documents and manuals for tourism statistics [1] nor the Encyclopedia of Tourism [2] offer an official definition of yachting tourism. Terms such as nautical tourism, marine or marina tourism, and leisure or recreational boating are commonly used as synonyms [3]. Yachting tourism should be considered part of the broader concept of nautical tourism [4], which is a particular form of tourism that uses watercraft or yachts for leisure activities; these include not only sailing, yacht cruising, rowing, kayaking, water skiing, and windsurfing activities, but also water-friendly activities such as surfing, fishing, swimming, recreational diving, and island exploration [5]. Yachting tourism in this paper is a narrow concept in China’s official “Yacht Safety Management Regulations,” which refer to vessels equipped with mechanical propulsion power for sightseeing, leisure, and other recreational activities. Previous research has focused on the economic, social, and ecological impacts of yachting tourism [6, 7], market segmentation [3], consumer behavior [8], and yachting tourism development strategies [9]. However, in the context of the increasing popularity of yachting tourism in China, few studies have examined safety issues in yachting tourism activities [10]. Since the safety of yachting tourism is related to the healthy development of the entire yachting tourism industry, this paper focuses on the risk factors affecting the safety of yachting tourism in coastal areas of China.

Safety is the prerequisite and primary purpose of all tourism activities. According to the United States (U.S.) Coast Guard’s Recreational Boating Statistics 2021, the U.S. Coast Guard recorded 4,439 accidents in 2021 in which 658 people died, 2,641 were injured, and recreational boating accidents caused approximately $67.5 million of property loss. According to the Royal National Lifeboat Institution in the United Kingdom, a total of 8,868 rescues took place in 2021, with an average of 35 people being rescued every day. In China, although the newly revised “Maritime Traffic Safety Law of the People’s Republic of China” and the “Yacht Safety Management Regulations” provide for the safety management of yacht tourism activities, the reality is that there are still a large number of safety accidents. For example, the China Cruise and Yacht Industry Association (CCYIA) collected insurance statistics for 1,335 yachts in recent years, and the percentage of accidents that occurred was as high as 6% [11]. In the face of growing yachting tourism activities, it is important to systematically research the safety risk assessment of yachting tourism and the corresponding management strategies.

Currently, researchers are focusing on assessing one element of yachting risk management [12]; for example, using principal component analysis (PCA) to determine how much personal behavior and normative beliefs affect how recreational boaters use buoys [13], or establishing the extent to which coastal weather parameters affect the safety of coastal marine activities [14]. Scholars are also investigating the availability of equipment such as lifeboats, rescue boats, and safety buoys at swimming beaches [15]; they assert that policies and regulations mandating the wearing of life jackets are important tools to promote safety and reduce drowning deaths [16, 17]. Requiring a minimum age for boat operators and offering boating courses in public schools have also had significant effects on reducing boating accidents [18]. In addition, some scholars have used a software called ArcGIS that uses the geographic information system (GIS) and cluster analysis to determine the distribution of marine tourism accidents in order to predict the high accident areas and common causes; they found that most accidents occurred during summer and peak tourist season due to mechanical problems caused by poor maintenance and carelessness [19].

Although previous studies help to identify the factors affecting yachting tourism safety and they provide specific management countermeasures for particular causes, they lack a comprehensive evaluation of the risk factors, which makes it difficult to identify key influencing factors and to propose targeted measures [20]. Further, in terms of research methodology, prior studies have used a large number of statistical and field survey methods; the results are mainly static which can only reflect the risk situation at a certain point in time and are not practical for dynamic risk analysis with environmental changes [21]. Thus, based on past studies, this paper introduces DBNs to evaluate YTSRs in coastal areas of China. This study not only comprehensively evaluated various risk factors, but also realized dynamic, ongoing evaluation of evolving characteristics over time.

In order to meet the research goals, this paper collected information on 115 cases of yachting tourism safety accidents in China’s coastal areas from 2008 to 2021, and used dynamic Bayesian theory to build and test a dynamic assessment model for YTSRs. First, YTSRs were identified through a fishbone diagram. Second, AHP was used to build an evaluation index system. Third, the risk of yachting tourism safety in coastal areas was divided into four quarters by employing DBNs. By multiplying the probability of safety risk events with potential losses, the dynamic evaluation outcomes of YTSRs were obtained. Countermeasures and suggestions for prevention and control of YTSR were then presented. The remainder of this paper is structured as follows: Section 2 contains a detailed literature review. Section 3 presents the primary research methods and data sources. Section 4 describes risk identification and indicator screening, as well as a dynamic evaluation model. Section 5 discusses the results. Finally, conclusions and insight are provided in Section 6.

## Literature review

Risk is a measure of uncertain outcomes and embodies the possibility of loss caused by risk factors to a disaster-bearing entity [22]. Tourism safety risks are possible, and the severity of risk-causing factors in tourism activities (which threaten tourists’ lives and property), as well as the process of screening and determining risk-causing factors, is a process of identifying sources of risk. The purpose of yachting tourism is recreation; not only the attributes of a boat, but also other water-based activities that involve yachts. Identifying risk factors includes four aspects: human, yachting, environmental, and management risk. Human factors are manifested in the occurrence of accidents due to defects in human psychological and physical qualities. The yachting factor entails the seaworthiness of the yacht itself and the configuration of its equipment. The environmental factor includes the meteorological and sea state environment, as well as the navigation context of yachting [23]. The management risk factor, which refers to the responsibility of each management subject, is not clear; there is cross management and the management blank phenomenon [24].

In terms of human factors, Lucrezi et al. used a structured questionnaire survey and collected data on diving centers and identified the risks affecting divers’ safety behavior from the level of safety awareness, including diving knowledge and attitudes towards the use of safety equipment [25]. As for yachting factors, Mentes and Helvacioglu used a design failure modes and effects analysis (DFMEA) to determine the failure elements, effects, and causes of yacht bilge water systems from the perspective of yacht system design [26]. Regarding environmental factors, Ferrari et al. defined beach hazards as elements of the beach and surf context that expose people to danger or injury, including rip currents, wave breaking, engineered structures, and slope stability [27]. Chen et al. identified the potential risks of coastal recreation activities as hydrodynamics and water quality in the surf zone based on two major categories of coastal recreation hazards summarized by the World Health Organization (WHO) [28]. Park et al. considered the marine environment to pinpoint the risks of yachting operations rooted in maritime traffic congestion [29]. As for management factors, Chen and Bau distinguished factors that affect beach safety from the standpoint of beach managers, including protective facilities and services provided by the beach [30]. These studies show that YTSRs are wide-ranging and require careful consideration of the degree of impact of different risks.

Researchers have primarily evaluated YTSRs based on two ways of thinking. First, the importance of risk factors is evaluated by calculating weights, and fuzzy hierarchical analysis (FHA) is the leading research technique. Chen and Bau used FHA to assess factors such as beach protection and management, facilities, and maintenance to identify priority factors and effective beach safety management [30]. Through FHA, Davila-Lamas et al. established the relative importance of parameters such as tide, temperature, wind speed, solar radiation, and bathymetry [31]. Davila-Lamas et al. synthesized all parameters via the fuzzy logic method to obtain a beach safety index to assess the safety of tourism activities in coastal areas [31]. This approach is grounded in fuzzy theory and is used to deal with uncertainty; it can also reduce decision-makers’ subjectivity so that accurate and significant factor weights can be found [32].

The second way is to estimate the hazard level of risk factors by calculating the probability of risk occurrence. Ferrari et al. determined the degree of coastal hazards by calculating a hazard index rooted in the basic principles of probabilistic theory for an appropriate combination of beach risk factors [27]. Xiao, Luo, and Li used Bayesian networks (BNs) to evaluate the safety risks of seaplane operation processes [20]. Ye et al. evaluated the risk of natural hazards in tourist areas by analyzing the exposure of tourism resources and tourism infrastructure, the vulnerability of residents and tourists, and computing the probability of occurrence of various risks and their potential losses [33]. These studies can identify key risk factors in a certain field, but the findings are mainly static, and scholars have been unable to carry out dynamic and continuous evaluation of risk factors over time.

YTSRs assessment is based on the identification of risk sources using a quantitative method to evaluate the possibility of different degrees of safety risks and their consequences. Yachting tourism has obvious seasonal characteristics. DBNs can consider uncertain information and risks, and make prediction and diagnostic analyses over time, which are widely used in the risk analysis of maritime accidents [34]. For example, Fan, Zheng, and Luo employed a BN model to examine the effectiveness of port state surveillance inspections and to gauge the level of ships’ safety at different times [35]. Jiang and Lu developed a DBN model using accident statistics and Markov chains to estimate dynamic contingency risks in marine waterways [36]. Khan et al. applied a Bayesian network risk assessment model by obtaining the prior probability distribution of ships in multiple locations in Arctic waters and performing a dynamic assessment of ship navigation risks [37]. Another study by Khan et al. used a dynamic Bayesian network method to estimate the dynamic collision risk of ship traffic in the Barents Sea [38]. The DBNs used in this paper not only integrates the advantages of static BNs, but also have a higher dynamic data processing capability [39], which can effectively reduce the uncertainty in the information fusion process and inference at different levels [40]. Hence, this paper introduced DBNs to achieve a dynamic and continuous safety risk evaluation of yachting tourism.

## Materials and methods

**Fig 1:**
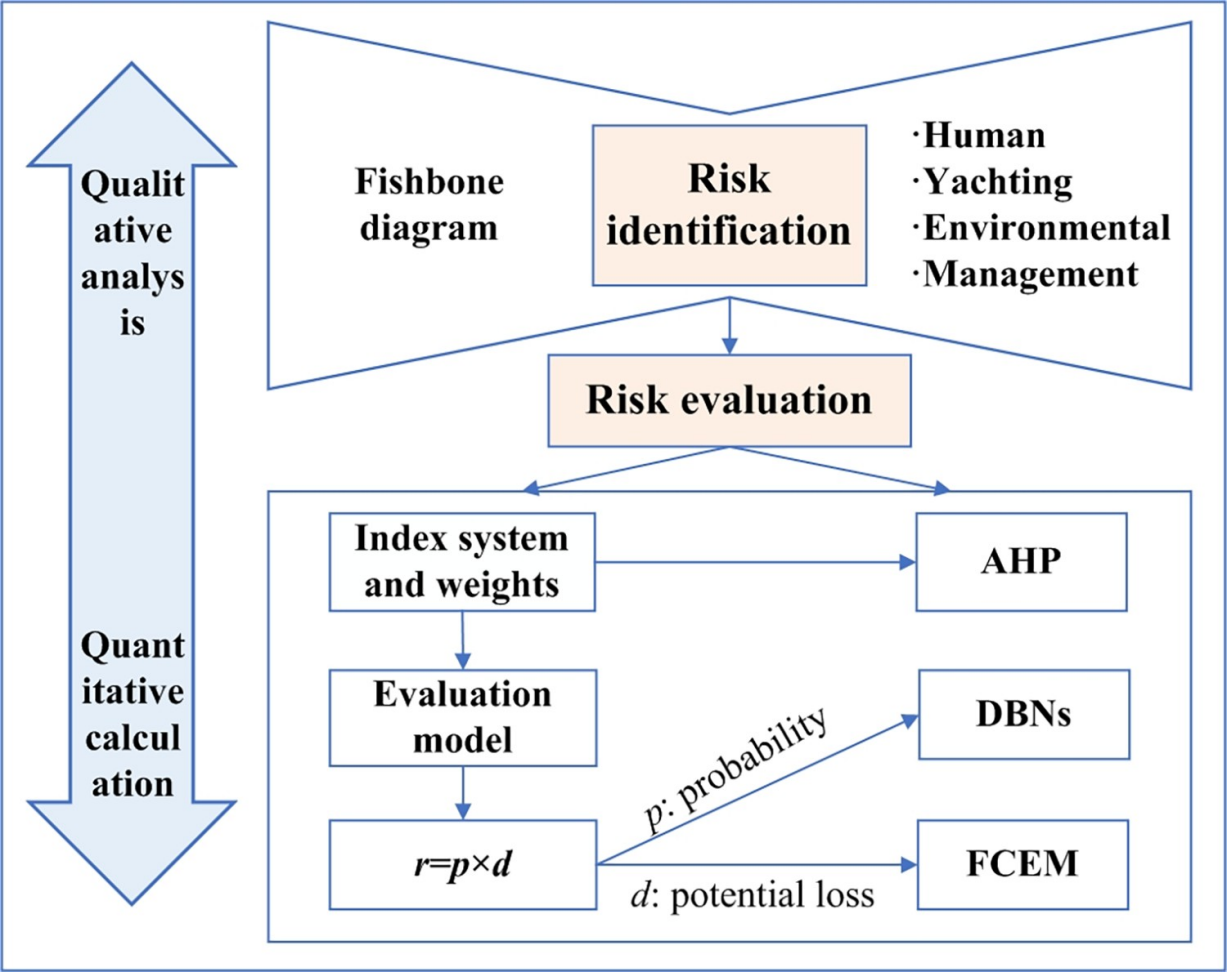
The method pattern of evaluation of YTSRs.

In this paper, a fishbone diagram and the AHP were used to identify and determine the security risk factors of yachting tourism. Further, DBNs and the fuzzy comprehensive evaluation method (FCEM) were employed to build a model to evaluate the security risks of yachting tourism in China’s coastal areas. The technical roadmap of the method is shown in Fig 1.

### Fishbone diagram

A fishbone diagram allows one to discover the root cause of a problem, representing the relationship between the outcome and the cause, and is a cause-effect analytical tool [41]. With the gradual in-depth study of influencing factors, a fishbone diagram can systematize the complex causes of accidents. We first analyzed the factors that have the greatest impact on the safety of yachting tourism, and then started from broader reasons to find out the medium-level reasons and small-level reasons. Finally, we detected and determined the main reasons.

### The AHP

AHP is suitable for organizing and analyzing complex decisions [42]; decomposing a decision problem into its components helps to establish a hierarchy of criteria to determine the importance of each indicator. The main steps for determining the weights of each indicator of the safety risk index system in yachting tourism are as follows: (i) The structure model of the AHP is established according to the relationship between the elements. (ii) Several experts are consulted to construct a pairwise judgment matrix of risk types. (iii) The hierarchical single and total ranking weights are calculated. Consistency checks are run and inconsistent acceptance is considered only when Consistency Ration (CR) < 0.1.

### FCEM

FCEM aims to determine the weight vector W through a statistical survey or hierarchical analysis, and to establish the affiliation degree of the risk evaluation index set C to the risk evaluation level set V by using the affiliation degree vector Q. Based on the evaluation index system, we calculated the potential loss of yachting tourism risk events using expert scoring and hierarchical analysis via a second-level FCEM. FCEM calculates risk loss in 5 key steps:

- The set of risk evaluation indicators *C* = {*c*_1_,*c*_2_,⋯,*c*_*n*_} is determined, and they are divided into several groups to obtain *C* = {*C*_1_,*C*_2_,⋯,*C*_*k*_}, which is called the first-level indicator set, and $C_{i}=\{c_{1}^{\left(i\right)},c_{2}^{\left(i\right)},\cdots ,c_{n_{i}}^{\left(i\right)}\}$ is called the second-level indicator set, among them $C=\overset{k}{\underset{i=1}{\cup }}C_{i}$.
- The evaluation grade set *V* = {*V*_1_,*V*_2_,…,*V*_*n*_} is established and expressed by the severity of yachting tourism risk events.
- The weight vector of each indicator is calculated through the AHP, and the weight of the second-level indicator set is $W_{i}=\{w_{1}^{\left(i\right)},w_{2}^{\left(i\right)},\cdots ,w_{n_{i}}^{\left(i\right)}\}$; the weight of the first-level indicator set is *W* = {*w*_1_,*w*_2_,⋯,*w*_*k*_}.
- First, the second-level index set is evaluated using expert scoring to obtain the single-factor evaluation matrix, which is $Q_{i}=[\begin{array}{cccc} q_{11}^{\left(i\right)}, & q_{12}^{\left(i\right)}, & \cdots , & q_{1m}^{\left(i\right)}\\ q_{21}^{\left(i\right)} & q_{22}^{\left(i\right)} & \cdots , & q_{2m}^{\left(i\right)}\\ \vdots & \vdots & \vdots & \vdots \\ q_{n_{i}1}^{\left(i\right)} & q_{n_{i}2}^{\left(i\right)} & \cdots & q_{n_{i}m}^{\left(i\right)} \end{array}]$; obtaining the fuzzy comprehensive evaluation produces *U*_*i*_ = *W*_*i*_×*Q*_*i*_(*i* = 1,2,⋯,*k*).
- Then, a fuzzy comprehensive evaluation is conducted on the first-level index set, and the overall evaluation matrix is $Q=[\begin{array}{c} Q_{1}\\ Q_{2}\\ \vdots \\ Q_{k} \end{array}]$. Obtaining the fuzzy comprehensive evaluation is *U* = *W*×*Q*, and the safety risk loss in yachting tourism works out by using the maximum membership principle.

### DBNs

DBNs are based on BNs, adding constraints of time attributes to form a network structure model that can reflect the evolution of events over time [43]. A BN model is shown in a directed acyclic graph formed by combining probability theory with graph theory, which contains a certain number of nodes and arcs [44], reflecting the causal dependencies between the nodes. For an edge connecting two nodes, the starting node is called a parent node and the ending node is called a child node. In BNs, nodes represent random variables, and directed arcs represent conditional dependencies between link nodes, which are described by dependent probability tables, and the dependent probability change process is consistent and stable for all time segments [45–47]. The time segment refers to a BN under different time intervals in a DBN, and the interval’s length depends on the event’s evolution. A DBN is a directed acyclic graph describing the probability relationship between time series variables; it consists of an initial network and a transition network.

For a BN graph, if the set of random variables is noted as *X* = {*X*_1_,*X*_2_,…,*X*_*n*_}, represents the corresponding node in the graph, *Pa*(*X*_*i*_) denotes the set of parents of node *X*_*i*_. *X*_*i*_ at the moment *t* is denoted as *X*_*i*_[*t*]. In Bayesian network theory, a BN is a directed acyclic graph containing the joint probability distribution over *X*. Each node *X*_*i*_ with the same parent *Pa*(*X*_*i*_) is conditionally independent of nodes that are not children of *X*_*i*_ from each other.

According to the basic principles of Bayesian network theory, a BN can be defined as: BN = (*G*, *θ*), where *G* is a directed acyclic graph of the joint probability distribution on *X*, and *θ* denotes the parameters of the network. The joint probability distribution on *X* is $P(X_{1},X_{2},\ldots ,X_{n})=\prod_{i=1}^{n}P(X_{i}|Pa(X_{i}))$.

A DBN model extends this formulation to model stochastic processes with a time factor. The DBN of the joint probability distribution, built on the time trajectory of the stochastic process, consists of two components: a priori net *B*_0_, with the joint probability distribution defined over the initial state *X*[1]; and a transfer network *B*_→_, defined by the transfer probability *P*(*X*[*t*+1]|*X*[*t*]) on the variables *X*[1] and *X*[2] (holds for all).

Thus, if a DBN model is given, the joint probability distribution is *X*[1],*X*[2],…,*X*[T] is $P(X[1],X[2],\ldots ,X[T])=P_{B_{0}}(X[1])\prod_{t=1}^{T}P_{B_{\to }}(X[t+1]/X[t])$. In the formula, *B*_0_ denotes the joint probability distribution over the initial state *X*[1]; *B*_→_ denotes the transfer network and *P*(*X*[*t*+1]/*X*[*t*]) indicates the transfer probability.

### Data source

We collected information on 115 cases of yachting tourism safety accidents in China’s coastal areas from 2008 to 2021. This sample size is consistent with the essential volume of yachting tourism safety accidents in coastal parts of China. For example, scholars have collected data on 920 marine tourism safety accidents in China’s coastal regions between 2010 and 2021, of which approximately 100 were related to yachting tourism [48]. Scholars have also assessed the safety risks of seaplane tourism using data from 110 seaplane accidents that occurred between 2010 and 2016 [20].

The case data are primarily derived from official reports and formal yachting websites, so they have a certain level of authenticity and dependability. The data sources include: (1) accident reports and related reports on yachts on the websites of government departments such as the China Maritime Service and the Ministry of Culture and Tourism; (2) accident reports and related news on portals tied to the yachting industry (such as https://www.yachter123.com/); (3) research papers and news reports about yachting tourism in literature databases such as the China Knowledge Network and the Wanfang Database, which we used to supplement the information. As for basic statistical information on the cases, regarding regional distribution, the Bohai Rim region accounted for 22.61%, the Yangtze River Delta accounted for 21.74%, the Pearl River Delta region accounted for 27.83%, and the Hainan province accounted for 27.83%; In terms of period distribution, the spring (March, April, May) accounted for 20%, the summer (June, July, August) accounted for 40%, the autumn (September, October, November) accounted for 21.74%, and the winter (December, January, February) accounted for 18.26%. The distribution in terms of region and time is more in line with the current status of Chinese yachting tourism activities.

## Results and discussion

We identified the risk influencing factors using the four elements of accident causation theory: (1) unsafe human behavior, (2) unsafe machines, (3) adverse environmental impact, and (4) lack of management [20, 49, 50]. A fishbone diagram can effectively analyze the significant, medium, minor, and lesser causes that endanger yachting tourism safety, and detect and identify important factors.

**Fig 2:**
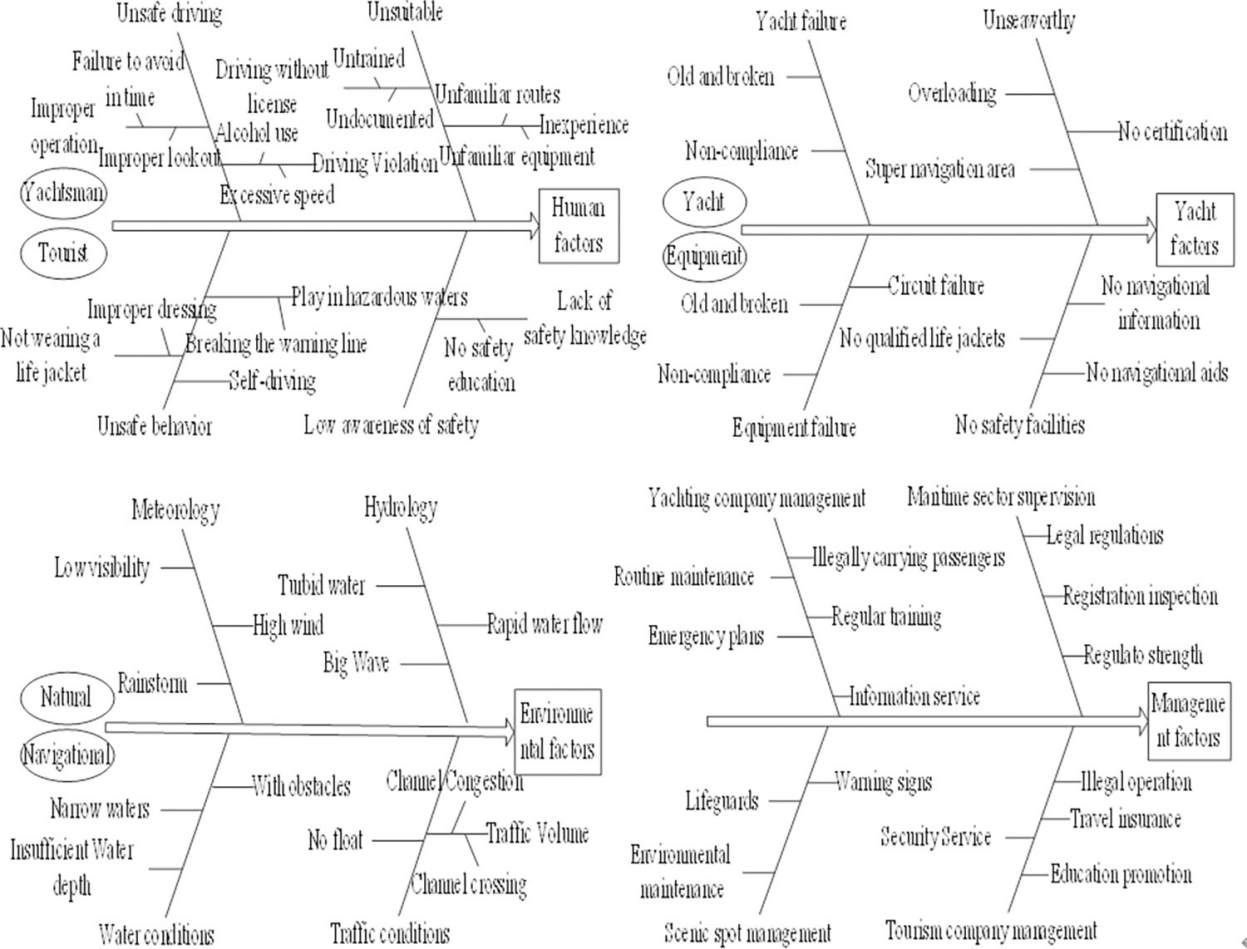
Fishbone diagrams for identifying safety risk factors for yachting tourism.

As shown in Fig 2, the risk of yachting tourism safety involves human, yachting, environmental, and management risk aspects, and involves a complex system of these four kinds of risks interacting. Human factors of yachting tourism include yachtsmen and tourists’ behavior and safety awareness. Yachting risk factors include the seaworthiness of the yacht and its equipment configuration. Environmental risk factors include the natural and navigable environment of yachting. Management risk factors include the registration and supervision of the yacht by the maritime department, as well as the daily supervision and maintenance of the yachting company. A yachting company refers to the yacht marina, club, or other yachting tourism core enterprises engaged in the operation and maintenance of yacht vessels. Tourism companies refers to enterprises engaged in yachting tourism operation services to recruit and receive tourists, providing sea itinerary arrangements and other business services.

Furthermore, the index system of safety risk evaluation in yachting tourism is built and empowered using scientific, systematic, targeted, and practical principles. From October 2021 to May 2022, we conducted field interviews and questionnaires with six experts from yachting ports/clubs, tourism enterprises, maritime departments, and research institutes. To calculate the weights of safety risk factors in yachting tourism, we used the AHP. First, we screened four significant causes and 16 medium causes to determine the framework of yachting tourism risk factors based on the identification order of the fishbone diagram and expert interviews. Second, we built a hierarchy for the identified risk factor framework, and we judged the relative importance of the selected factors through pairwise comparison of the questionnaire. The index system of safety risk evaluation in yachting tourism and the weights are shown in Table 1, which provides the theoretical basis for the subsequent safety risk evaluation for yachting tourism based on DBNs.

**Table 1:** Yachting tourism safety risk evaluation index system and weights.

First level	Second level	Weights	Third level	Weights	Synthetic weights	Sort
C: Safety risk of yachting tourism	C1: Human factors	0.467	C11: Unsafe driving	0.32	0.149	3
			C12: Unsuitable	0.146	0.068	6
			C13: Unsafe behavior	0.472	0.22	1
			C14: Low awareness of safety	0.062	0.029	11
	C2: Yacht factors	0.277	C21: Yacht failure	0.066	0.018	12
			C22: Unseaworthy	0.604	0.167	2
			C23: Equipment failure	0.119	0.033	9
			C24: No safety facilities	0.211	0.058	7
	C3: Environmental factors	0.095	C31: Meteorology	0.513	0.142	4
			C32: Hydrology	0.314	0.03	10
			C33: Water conditions	0.058	0.006	16
			C34: Traffic conditions	0.115	0.011	14
	C4: Management factors	0.16	C41: Yachting company management	0.528	0.084	5
			C42: Maritime sector supervision	0.347	0.056	8
			C43: Scenic spot management	0.076	0.012	13
			C44: Tourism company management	0.05	0.008	15

A risk of yachting tourism safety is the probability of a hazardous event occurring during a specific period for yachting tourism and the potential loss it causes. Therefore, the quantitative risk calculation method applied in this paper is *r* = *p*×*d*.

In the form, *r* is the risk value of the event, *p* is the probability of the hazardous event occurring, and *d* is the consequence of the accident as the potential loss. In this paper, the potential loss refers to the number of casualties and property damage in yachting tourism activities. The DBN model determines the probability of hazardous events in yachting tourism. The potential loss can be obtained through accident statistical analysis and FCEM [51]. As shown in Table 2, we refer to the accident class classification criteria in the Statistical Approach to Water Traffic Accidents (2021), and we determined the severity of hazardous events based on risk-consequence guidelines, expert surveys, and scholarly studies [52].

**Table 2:** Classification of the severity of hazardous events.

Level	Severity description
Minor	Minor injuries, resulting in direct economic losses ranging from 10 to 50,000 yuan.
Serious	Serious injury, causing direct economic losses of 50,000–100,000 yuan
More serious	1~2 deaths, causing direct economic losses of 100,000–300,000 yuan
Very serious	3~9 deaths, causing direct economic losses of 300,000–500,000 yuan
Fatal	More than 10 deaths, resulting in direct economic losses of more than 500,000 yuan.

**Fig 3:**
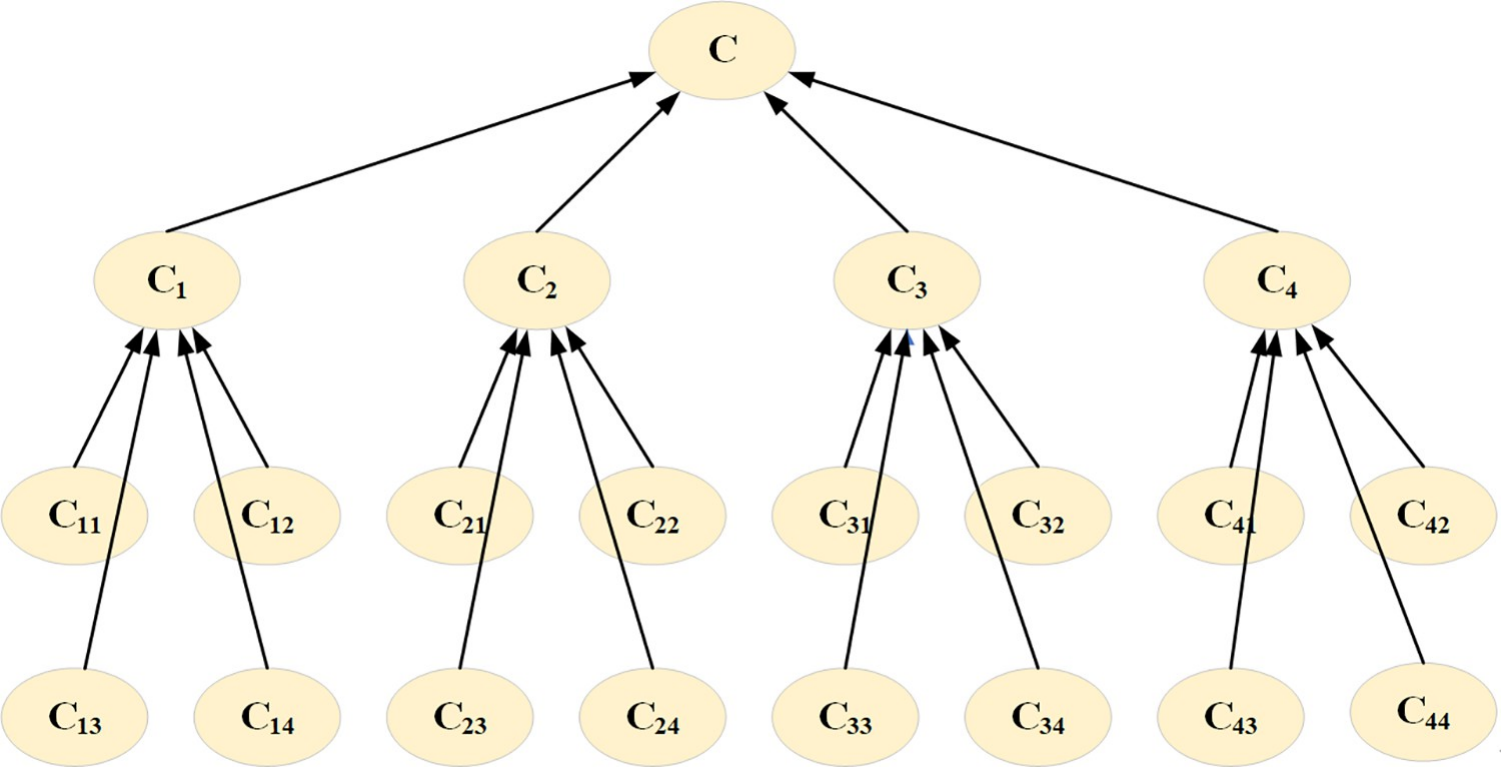
The Bayesian network structure of yachting tourism.

The construction of a DBN includes the structure and parameter aspects. In this paper we obtain the structure according to the 3-level indicator system [20], and we obtained the parameters using both expert experience and the expectation-maximization (EM) algorithm [53]. The specific steps are as follows:

The node variables are determined. A Bayesian network structure was constructed based on the results of the identification and screening of risk factors (Fig 3). The BN structure has 16 root nodes, four intermediate nodes, and one leaf node. The single-leaf node (C) represents a yachting tourism safety accident. According to the classification standard of accident level above, the status of yachting tourism safety accident (C) is set as “minor,” “serious,” “more serious,” “very serious,” and “fatal” to describe the severity of the accident. The occurrence of safety accidents in yachting tourism involves four risks: human (C1), yachting (C2), environment (C3), and management risk (C4), each of which has a “yes” and “no” status. A “yes” status indicates that the accident was related to this risk, while a “no” means it was not. The four main risks are, in turn, made up of 16 fundamental risk events (C11, C12,……). These events are denoted by root nodes with two states, where “yes” means that a risk event has occurred, and “no” means that no risk event has occurred.

**Fig 4:**
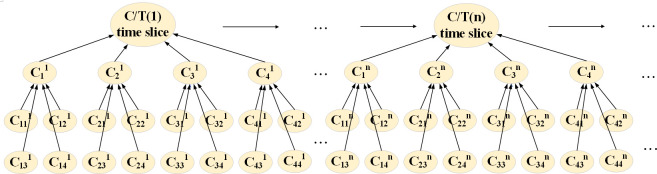
The DBN structure of yachting tourism.

The network topology is determined. A DBN is formed by adding time slices to the BN base. The human geographic environment in yachting tourism is complex, and the characteristics of seasonal changes are apparent. The nodal variables present different states on different time slices, eventually forming a dynamic structure that changes over time. As seen in Fig 4, C/T(1) represents the state of the yachting tourism safety incident at the first time slice and C/T(n) indicates the state at the nth time slice.

The conditional probabilities of significant nodes are obtained. The DBN parameters were obtained through two steps. First, the prior probability table and the conditional probability table were given directly by the same six experts mentioned above. The problem scope, the elicitation process, and the fundamental theory of DBN were introduced in detail to each expert to guarantee that they understood the objective of this process [54]. A consensus on the DBN parameters was reached. The next step was parameter learning. We input the 115 yachting tourism accidents that took place from 2008 to 2016 into the parameter learning EM algorithm to obtain the final prior probability table and the conditional probability table. Without the first step, parameter learning can also work, but the expert experience in the first step provides the prior knowledge needed for parameter learning, and thus makes the DBN model more accurate and practical.

**Fig 5:**
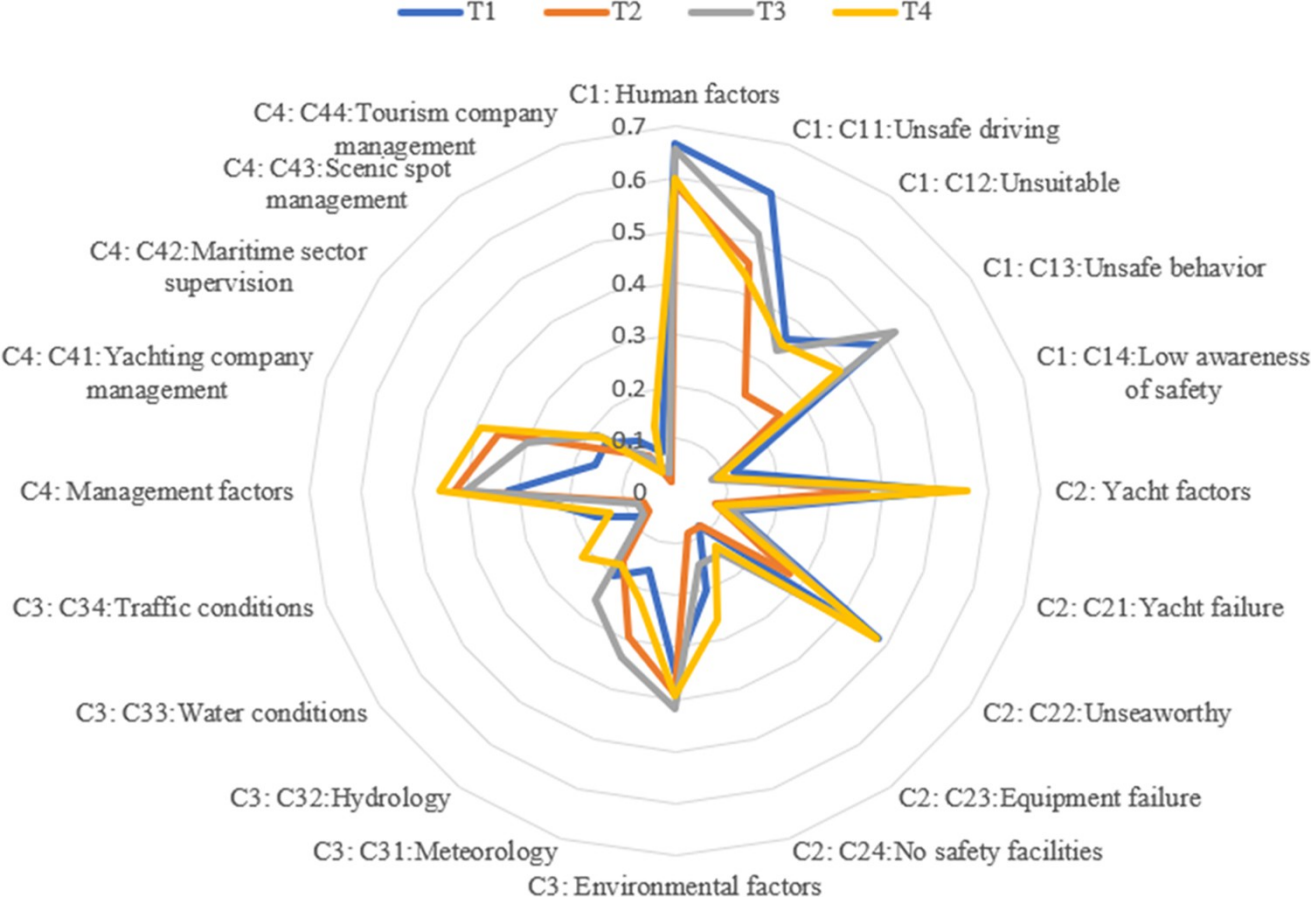
Changes in the probability of risk factors occurring in the first quarter (T1), second quarter (T2), third quarter (T3), and fourth quarter (T4).

The results are calculated. Based on the results and parameters of the DBN model, the probability of hazardous events in yachting tourism (Table 3) and the likelihood of its risk factors (Fig 5) were calculated using Netica 5.18. Based on the four busy periods divided, the first time slice is T (March, April, May); the next time slice is T+1, and so on.

**Table 3:** The probability of a hazard occurring.

Severity	T1	T2	T3	T4
Minor	0.213	0.191	0.195	0.196
Serious	0.157	0.202	0.204	0.192
More serious	0.276	0.225	0.211	0.221
Very serious	0.176	0.190	0.200	0.196
Fatal	0.178	0.192	0.190	0.196

Note: "T1" indicates the spring (March, April, May); "T2" indicates the summer (June, July, August); "T3" indicates the autumn (September, October, November); "T4" indicates the winter (December, January and February).

Among the four time films (see Fig 5), the probability of human factors is the highest, all reaching about 0.6, of which unsafe driving and unsafe behavioral factors play the most prominent roles, with the probability being about 0.5. In addition to human factors, the probability of yachting factors is the highest at T1, T3 and T4, all of which are more than 0.5. The unseaworthy factor is the most obvious, and the probability is about 0.4. The probability of management factors at T2 is the highest, which is 0.423; among them, the yachting company management plays the most prominent role, and the probability is 0.354. Thus, human factors are the primary risk factors, mainly unsafe driving and unsafe behavior factors. Yachting factors mostly unseaworthy factors, have the second greatest impact. Management risk factors primarily yachting company management factors, have the third greatest impact. Environmental factors have the least impact.

Using the equal spacing method, we further classified the safety risk level for yachting tourism into four levels low, medium, higher, and increased risk [47, 55] as shown in Table 4.

**Table 4:** Results of the dynamic evaluation of YTSRs.

Risk level	Lower risk	Medium risk	Higher risk	Great risk
Risk value	0.041~0.055	0.056~0.070	0.071~0.085	>0.086

There are obvious differences in the spatial and temporal distribution of YTSRs, and the risk levels in different regions are constantly shifting with the change of seasons (see Table 5). First, in the Bohai Rim region, the risk value in summer reaches 0.073, which is a “higher risk”, while in other seasons the risk value is below 0.055, which is a “lower risk”. The Bohai Rim region is only at greater risk in summer and lower risk in other seasons. This is due to the construction of infrastructure, such as yacht marinas and berths around the Bohai Rim region behind other regions [11], as well as the temperate monsoon climate. The winter temperature is low, below 0°C [56], which is not suitable for recreational water activities. Second, in the Yangtze River Delta region, the risk values change most significantly throughout the year, from high to low, in order of 0.098, 0.076, 0.057 and 0.052, reflecting risks in spring, autumn, summer and winter respectively. The Yangtze River Delta has abundant resources and perfect infrastructure that attracts many yachting tourists. It is during the spring travel season that the number of yachting tourists reaches its peak and the risk is greatest. Third, the risk value of the Pearl River Delta region reaches 0.071 in summer, which is "higher risk", and the risk value of Hainan region reaches 0.09, which is " great risk" in winter. These areas are "medium risk" during other seasons, when the risk value is between 0.056 and 0.070. The Pearl River Delta and Hainan regions are at medium and high risk all year round, mostly due to their low latitude, mild climate in all seasons, rich marine tourism resources, and perfect yacht infrastructure [11].

**Table 5:** Results of the dynamic evaluation of YTSRs in China.

Region	Spring	Summer	Autumn	Winter
The Bohai Rim Region	0.049	0.073	0.055	0.041
The Yangtze River Delta Region	0.098	0.057	0.076	0.052
The Pearl River Delta Region	0.058	0.071	0.057	0.06
Hainan Province	0.057	0.064	0.061	0.09

## Conclusions

At present, the research on safety risk assessment in yachting tourism is primarily based on identifying sources of risk, which are only evaluated from the aspects of human, yachting, environmental, and management risk factors. As for research methods, research has only evaluated the importance of risk factors by calculating weight, or estimating the degree of harm of risk factors by calculating probability. However, few studies have comprehensively assessed the four factors of human, yachting, environmental, and management risk factors from a holistic perspective. The evaluation results are mainly static and can only reflect the risk situation at a certain point in time. We comprehensively evaluated four risk factors human, yachting, environmental, and management risk—by means of the AHP and DBNs, and we identified the chief risk factors affecting yachting tourism safety. At the same time, we performed a dynamic evaluation of YTSRs. Our main conclusions are as follows.

First, we discovered that China’s YTSRs are complex and diverse, primarily consisting of four major risk categories: human, yachting, environmental, and management risk. Each risk category contains four risk sub-categories. Human risks include unsafe behavior and lack of awareness among drivers and tourists. Yachting risks include the seaworthiness of the yacht and its equipment configuration. Environmental risks include the natural environment and the navigable environment. Management risks are mostly concerned with the situation of the four management bodies of yachting tourism. A YTSRs is formed by the mutual influence and interaction of these four types of risks, and effectively dealing with the relationship between them is key to preventing and controlling risks [49].

Second, there are noticeable spatial and temporal distribution differences in the safety risks of Chinese yachting tourism, with greater risks in the south than the north, and higher risks in summer versus winter. Therefore, it is necessary to dynamically prevent and control risks based on time-sharing, zoning, and situational changes [28].

Third, the primary safety risk factors for yachting tourism activities are human factors, such as yachtsmen [25]. Yachting tourism is not only a tourism activity, but also a leisurely sport, so we should improve the skills training of yachting tourism practitioners, popularize yachting safety knowledge, and raise tourist safety awareness.

The control of yachting tourism safety risks is a complex project that requires the active cooperation of all parties. We therefore offer the following recommendations: (1) The transportation and maritime departments should improve the specific rules and regulations of yacht registration, inspection, navigation, and safety that do not correspond to the current actual situation, and differentiate yachting tourism management from other types of ship management. The Ministry of Culture and Tourism, marine fisheries, meteorology, and other departments must actively collaborate in terms of tourism market safety supervision and risk prediction. At the same time, due to the different types of safety risks of yachting tourism (risk management has cross-regional, cross-departmental and decentralized monitoring characteristics), it is necessary to strengthen the deployment of rescue and security resources in high-risk areas. It is important to rely on big data and electronic information technology to create an intelligent command and control platform for yachting tourism in high-risk time zones and to achieve dynamic monitoring of illegal yachting. (2) To ensure the safe development of yachting tourism activities, yachting clubs and other enterprises must improve their management capabilities, strengthen daily supervision of yachts and ship maintenance, ensure safety skills training for yacht drivers, distribute and regular publicity on yacht safety and anti-pollution knowledge. (3) Tourism enterprises and coastal scenic spots should be provided to tourists for safety and services, such as installing warning signs in dangerous waters and assisting with life-saving personnel and equipment, as well as providing yachting tourism information and consultation, travel insurance, and other services. (4) Furthermore, tourists should take the initiative to learn about yacht safety and avoid risky behaviors while yachting. In particular, self-driving tourists should ensure that they are proficient at driving and can save themselves and help each other.

To date, researchers have developed a variety of tourism safety risk assessment methods. The Fuzzy Analytical Hierarchy (FHA) method is often used to calculate the risk index affecting the safety risk factors of yachting tourism [30, 31]. The disadvantage of this method is the subjectivity of the evaluation result and problems in dealing with uncertain information in the analysis [34]. Fault Tree Analysis (FTA) [57, 58] is often used to calculate the probability of maritime accident risk factors, but its evaluation results cannot reflect real-time dynamic information [59]. The dynamic evaluation model of YTSRs proposed in this paper can predict and diagnose YTSRs over time. This model can not only to identify the main risk factors, but also to reflect the probability of occurrence of each risk factor in different periods and thus to provide dynamic evaluation results. Therefore, this study not only enriched and expanded the content and methods of yachting tourism research, but also provided stakeholders with practical guidance for the formulation of policies and plans to effectively control and reduce risks in the development of yachting tourism.

However, this study is not without its limitations. Firstly, the size of the data on yachting tourism accidents is relatively small due to a lack of public government statistics, and the information on each accident can only reflect the casualties and a portion of the property damage. The information does not fully account for the harm done to other tourism facilities and marine pollution. As a result, the risk evaluation model only considers the number of casualties and property damage. The accuracy of the evaluation outcomes could be improved in subsequent research by increasing accident data and collecting comprehensive accident information. Secondly, we used DBNs to calculate the probability of safety risk factors in yachting tourism at different times, but their sensitivity has not been analyzed. This is because the probability gap of each risk factor at different times is small; it is of little significance to analyze the sensitivity, and the sensitivity analysis of a DBN requires introducing special algorithms with high computational complexity [60]. Hence, future research can deepen the learning of DBNs on the premise of collecting a large sample of data, and identify key risk factors using diagnostic reasoning and sensitivity analysis of DBNs, in order to provide scientific support for relevant departments so they can formulate effective security risk prevention and control policies.

## Supporting information

S1 File Case information statistics. (XLSX)
